# Supplementary material for: Synergistic Photodynamic/Antibiotic Therapy with Photosensitive MOF-Based Nanoparticles to Eradicate Bacterial Biofilms
Source: Pharmaceutics. 2023 Jun 26;15(7):1826. doi: 10.3390/pharmaceutics15071826 (PMC10385796; doi:10.3390/pharmaceutics15071826)
Supplement: Supplementary file 1 [file pharmaceutics-15-01826-s001.zip › pharmaceutics-2448626-supplementary.pdf]

pharmaceutics

MDPI

Article

# Synergistic Photodynamic/Antibiotic Therapy with Photosensitive MOF-based Nanoparticles to Eradicate Bacterial Biofilms

Lehan Du [1], Wenjun Shi [1], Xin Hao [1], Liang Luan [2,*], Shibo Wang [1], Jiaju Lu [1] and Quan Zhang [1,*]

[1] Institute of Smart Biomedical Materials, School of Materials Science and Engineering, Zhejiang Sci-Tech University, Hangzhou 310018, China
[2] Department of Laboratory Medical Center, General Hospital of Northern Theater Command, No.83, Wenhua Road, Shenhe District, Shenyang 110016, China
* Correspondence: liang_luan@163.com (L.L.); quanzhang@zstu.edu.cn (Q.Z.)

**Citation:** Du, L.; Shi, W.; Hao, X.; Luan, L.; Wang, S.; Lu, J.; Zhang, Q. Synergistic Photodynamic/Antibiotic Therapy with Photosensitive MOF-Based Nanoparticles to Eradicate Bacterial Biofilms. *Pharmaceutics* **2023**, *15*, x. https://doi.org/10.3390/xxxxx

Academic Editor: Maria Nowakowska

Received: 29 May 2023
Revised: 20 June 2023
Accepted: 22 June 2023
Published: 26 June 2023

**Copyright:** © 2023 by the authors. Submitted for possible open access publication under the terms and conditions of the Creative Commons Attribution (CC BY) license (https://creativecommons.org/licenses/by/4.0/).

## 1. Experimental Section

**Materials.** Zirconium(IV) chloride ($ZrCl_4$, 98%), polymyxin B sulfate salt (PMB, ≥6000u/mg), tween 80 (99.5%), benzoic acid (99%), 1-(3-dimethylaminopropyl)-3-ethylcarbodiimide (hydrochloride) (EDC · HCl, 97%), N-hydroxy succinimide (NHS, 98%), N, N-dimethylformamide (DMF, 99.8%), ethanol (EtOH, 99.5%), methyl alcohol (MeOH, 99.5%) were purchased from Shanghai Titan Scientific Co., Ltd. Phosphate-buffered saline (PBS) was purchased from Cytiva Inc. (China). Yeast extract and tryptone were obtained from Thermo Fisher Scientific. Agar power was purchased from Shanghai EKEAR BioNTech Co., Ltd. Crystal violet staining solution (≥1 - ≤2.5%), propidium iodide (PI, ≥94.0%) and ROS Assay Kit (DCFH-DA, 10 mM) were purchased from Beyotime Inc. (China). SYTO 9/PI Live/Dead Bacterial Double Stain Kit (SYTO9, 3.34 mM; PI, 20 mM) was purchased from Shanghai Maokang Biotechnology Co., Ltd. All other chemicals were commercially available and used without further purifications.

**General Characterizations.** Scanning electron microscopy (SEM) images were produced using a vltra55 scanning electron microscope (CarlZeissSMTPteLtd), which operated at 3.0 KV accelerating voltage. Transmission electron microscopy (TEM) images were acquired using a JEM-1400Flash transmission electron microscope operated at 120 KV. Powder X-ray diffraction (XRD) analyses were performed and XRD patterns were recorded on a Bruker A8 Advance X-ray diffractometer using monochromatic Cu K$\alpha$ radiation ($\lambda$ = 1.5406 Å) at room temperature. The electron spin resonance (ESR) spectra were recorded using a Brucker A300 spectrometer at room temperature. The hydrodynamic size and zeta potential measurements were performed using a Malvern Zetasizer NanoZS instrument at 25 °C. UV-vis absorption spectra were recorded on a P7 Double Beam UV-vis spectrometer (China). Fourier-transform infrared spectroscopy (FT-IR) spectra were obtained as KBr-pellets on a Nicolet iS50 FT-IR spectrometer. Confocal microscopy images (CLSM) were captured using a Nikon A1 confocal microscope. Flow cytometry analyses were performed using an Agilent NovoCyte Advanteon flow cytometer. Light irradiance was performed using a CEL-NP2000-2(10)A fully automatic optical power meter.

The results were presented as mean ± standard deviation (SD). Data were analyzed for statistical significant differences (* $p < 0.05$, ** $p < 0.01$, and *** $p < 0.001$) using the SPSS software.

**2. TEM images of Se-NPs**

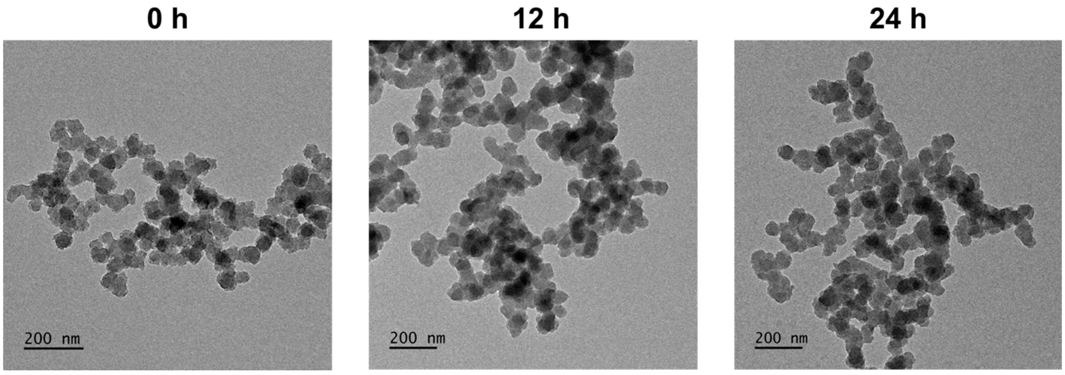


**Figure S1**. TEM images of Se-NPs after incubation in LB media for different times.

**3. Emission spectrum and characteristics of LED lamps**

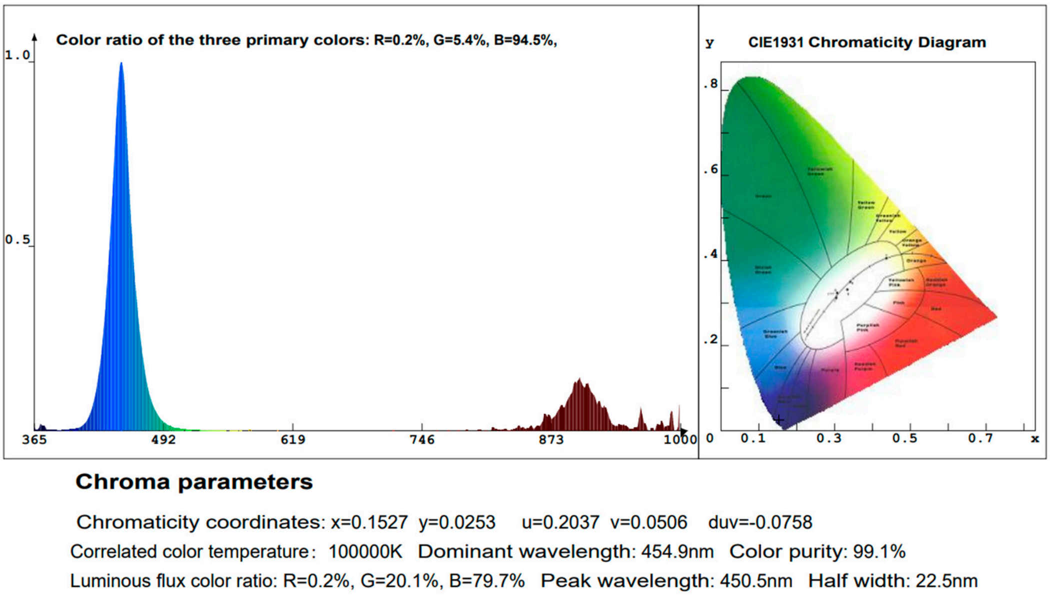


**Figure S2**. Emission spectrum and characteristics of the LED light.

## 4. TEM image of PMB-Se-NPs

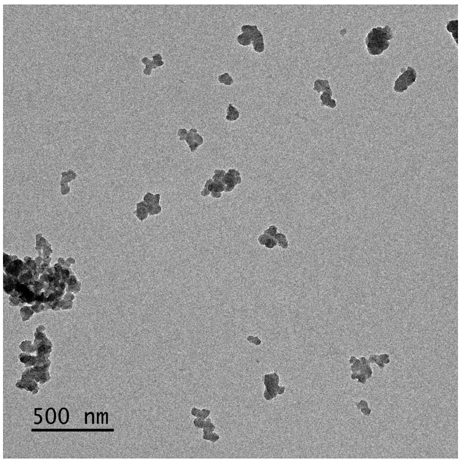


**Figure S3**. TEM image of PMB-Se-NPs.

## 5. Standard curve of PMB

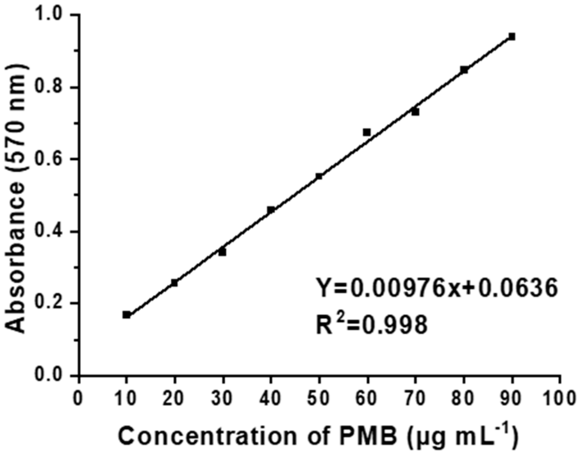


**Figure S4**. The standard curve of absorbance vs. PMB concentration.

## 6. Fluorescence emission spectra of Se-NPs, PMB, and PMB-Se-NPs

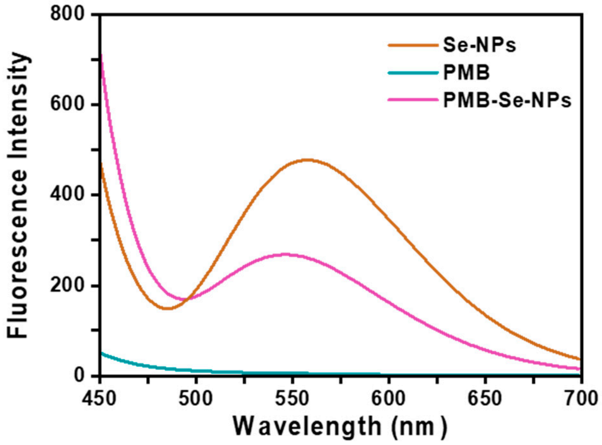


**Figure S5.** Fluorescence emission spectra (λex = 390 nm) of Se-NPs, PMB, and PMB-Se-NPs dispersed in PBS buffer.

## 7. Evaluation of *P. aeruginosa* biofilm formation by crystal violet staining

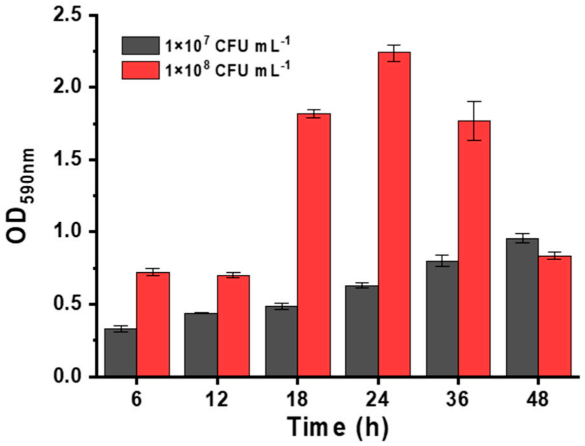


**Figure S6.** Crystal violet staining results of *P. aeruginosa* biofilm after different bacterial concentrations and culture times.
